# An Integrative Phenotype–Genotype Approach Using Phenotypic Characteristics from the UAE National Diabetes Study Identifies *HSD17B12* as a Candidate Gene for Obesity and Type 2 Diabetes

**DOI:** 10.3390/genes11040461

**Published:** 2020-04-23

**Authors:** Mahmood Y. Hachim, Hayat Aljaibeji, Rifat A. Hamoudi, Ibrahim Y. Hachim, Noha M. Elemam, Abdul Khader Mohammed, Albert Salehi, Jalal Taneera, Nabil Sulaiman

**Affiliations:** 1 Sharjah Institute for Medical Research, University of Sharjah, Sharjah 27273, UAE; U16101425@sharjah.ac.ae (M.Y.H.); hayat_aljaibeji@yahoo.com (H.A.); rhamoudi@sharjah.ac.ae (R.A.H.); noha.elemam211@gmail.com (N.M.E.); amohammed@sharjah.ac.ae (A.K.M.); 2 Department of Clinical Sciences, College of Medicine, University of Sharjah, Sharjah 27272, UAE; ihachim@sharjah.ac.ae; 3 Department of Clinical Sciences, Division of Islets Cell Pathology, Lund University, SE-205 02 Malmö, Sweden; s_albert.salehi@med.lu.se; 4 Department of Basic Medical Sciences, College of Medicine, University of Sharjah, Sharjah 27272, UAE; 5 Department of Family and Community Medicine and Behavioral Sciences, College of Medicine, University of Sharjah, Sharjah 27272, UAE; 6 Baker Heart and Diabetes Institute, Melbourne 3004, Australia

**Keywords:** GWAS, diabetes, biomarkers, SNPs

## Abstract

The United Arab Emirates National Diabetes and Lifestyle Study (UAEDIAB) has identified obesity, hypertension, obstructive sleep apnea, and dyslipidemia as common phenotypic characteristics correlated with diabetes mellitus status. As these phenotypes are usually linked with genetic variants, we hypothesized that these phenotypes share single nucleotide polymorphism (SNP)-clusters that can be used to identify causal genes for diabetes. We explored the National Human Genome Research Institute-European Bioinformatics Institute Catalog of Published Genome-Wide Association Studies (NHGRI-EBI GWAS) to list SNPs with documented association with the UAEDIAB-phenotypes as well as diabetes. The shared chromosomal regions affected by SNPs were identified, intersected, and searched for Enriched Ontology Clustering. The potential SNP-clusters were validated using targeted DNA next-generation sequencing (NGS) in two Emirati diabetic patients. RNA sequencing from human pancreatic islets was used to study the expression of identified genes in diabetic and non-diabetic donors. Eight chromosomal regions containing 46 SNPs were identified in at least four out of the five UAEDIAB-phenotypes. A list of 34 genes was shown to be affected by those SNPs. Targeted NGS from two Emirati patients confirmed that the identified genes have similar SNP-clusters. *ASAH1*, *LRP4*, *FES*, and *HSD17B12* genes showed the highest SNPs rate among the identified genes. RNA-seq analysis revealed high expression levels of *HSD17B12* in human islets and to be upregulated in type 2 diabetes (T2D) donors. Our integrative phenotype-genotype approach is a novel, simple, and powerful tool to identify clinically relevant potential biomarkers in diabetes. *HSD17B12* is a novel candidate gene for pancreatic β-cell function.

## 1. Introduction

Type 2 diabetes (T2D) is a multifactorial disorder characterized by the insufficiency of insulin secretion and/or insulin action [1]. The disease is one of the most critical public health challenges of the 21st century [2]. Rapid socio-economic transition is one of the leading causes of the global diabetes epidemic and rising prevalence rates in developing economies [3]. The cost associated with the disease affects more impoverished world regions as well as high-income countries, thus imposing a substantial global economic burden [4]. Recent reports showed a high prevalence of T2D in the United Arab Emirates (UAE) [5]. Thus, effective interventions are urgently needed to slow the diabetes epidemic and reduce diabetes-related complications. An ultimate goal in diabetes management is the identification of novel biomarkers that enable the detection, prevention, treatment of the disease, and its complications long before overt disease development [6].

Genome-wide association studies (GWAS) have identified more than 143 common genetic variants associated with T2D [7]. However, these variants explain only a small proportion of the heritability of the disease [8]. Thus, more extensive studies are needed to identify T2D loci in different populations, as well as novel approaches to make sense of these generated databases [9].

The United Arab Emirates National Diabetes and Lifestyle Study (UAEDIAB) study is a cross-sectional survey designed to investigate the prevalence of diabetes and associated risk factors in 827 Emiratis and 2724 expatriates living in Dubai, Sharjah, and the Northern Emirates [10,11,12]. UAEDIAB study has identified a list of patient’s related measurements including obesity/BMI, hypertension, obstructive sleep apnea, and dyslipidemia that correlated with diabetes. Interestingly, most of these identified phenotypes have previously been associated with genetic variants [13,14,15,16].

Random genetic variants are unlikely to occur in the same genomic position unless there is a positive selection for that mutation, which might represent a fitness advantage [17]. However, genetic variants in terms of single nucleotide polymorphism (SNP) can cluster in particular chromosomal regions [18]. Those regions might represent an increased vulnerability to mutation as a result of their unique sequence, structural, and functional features [19]. Such SNPs clusters are thought to occur preferentially around some functional genes. Therefore, the identification of clustered SNPs might provide insights into the molecular mechanisms of mutagenesis. This may include vulnerable regions, possible positive selection for disease development, and regulatory actions associated with T2D.

In this study, we describe an integrative phenotype-genotype approach to identify novel potential biomarkers for T2D by utilizing the phenotypic characteristics of the UAEDIAB study. This was addressed by a systems genetics approach integrating SNP-clusters in chromosomal regions between the identified UAEDIAB-phenotypes and published SNPs using the National Human Genome Research Institute-European Bioinformatics Institute Catalog of Published Genome-Wide Association Studies (NHGRI-EBI GWAS) Catalog. We believe that our study might lead to novel testable hypotheses for genes involved in β-cell function and will add new insight into the molecular pathogenesis and biomarkers for T2D.

## 2. Materials and Methods

### 2.1. Study Design, Population, and Settings

The details of the study design and sampling of the UAEDIAB study are described elsewhere [10,20]. In brief, the UAEDIAB is a cross-sectional study conducted to estimate both prevalence and risk factors of diabetes among UAE nationals and expatriates who have been living in Sharjah, Dubai, and the Northern Emirates for at least four years. The study was approved by the Ethics Committee of Sharjah University and the Ministry of Health Research Ethics Committee (REC number MOHAP/DXB/SUBC/No.14/2017). All participants had consented to the usage of their collected data and samples.

### 2.2. Identification of SNP-Clusters Associated with the UAEDIAB-Phenotypes

We utilized the NHGRI-EBI GWAS Catalog (https://www.ebi.ac.uk/gwas/, v. 1.0) of published genome-wide association studies [21]. The file downloaded was (gwas_catalog_v1.0-associations_e96_r2019-07-12). The entire catalog was downloaded and prioritized for SNPs associated with the 5 given phenotypes; (1) diabetes (type 1 diabetes (T1D) and T2D), (2) obesity (obesity and body mass index), (3) dyslipidemia (triglyceride levels, triglycerides, lipid traits, high-density lipoprotein (HDL) cholesterol-triglycerides (HDLC-TG), low-density lipoprotein (LDL) cholesterol levels, HDL cholesterol levels, and total cholesterol levels), (4) obstructive sleep apnea (snoring, obstructive sleep apnea, obstructive sleep apnea trait (apnea–hypopnea index), (average oxygen saturation during sleep), or (average respiratory event duration), and (5) hypertension (systolic blood pressure ≥ 140/diastolic blood pressure ≥ 90. SNPs with a significant association with each of the given phenotypes associated with diabetes (adjusted *p*-value < 0.05) were identified along with their corresponding chromosomal regions.

### 2.3. SNP to SNP Functional and Pathway Enrichment

To investigate whether the identified genes share common pathways that may link the given phenotypes, we used eXploring Genomic Relations (XGR) for enhanced interpretation web tools (http://galahad.well.ox.ac.uk:3040/). XGR provides enhanced interpretation of GWAS via comprehensively utilizing ontology and network information [22]. The tool uses the Experimental Factor Ontology (EFO) option to provide a systematic description of many experimental variables available in EBI databases and the NHGRI-EBI Catalog [23]. Enrichment analysis is based on the hypergeometric/binomial distribution or Fisher′s exact test which tests the statistical significance of the observed number of SNPs overlapped between an input group of SNPs and SNPs annotated by EFO. As validation of the results, Enriched Ontology Clustering for the identified genes and SNPs associated with the 5 identified UAEDIAB-phenotypes study were generated using the Metascape (a web-based tool used for comprehensive gene list annotation and analysis resource) and Reactome option in SNPnexus [24]. Socialiser for SNPs in XGR was used to assess the degree of relatedness between any two SNPs in terms of annotation profiles and network were performed by the tool.

Similarity functions serve to conduct similarity analysis calculating semantic similarity—a type of comparison to assess the degree of relatedness between two entities (e.g., genes or SNPs) based on their annotation profiles (by ontology terms) [25]. To do so, information content (IC) of a term is first defined to measure how informative a term is to being used for annotating genes: −log10 (frequency of genes annotated to this term). The similarity between the two terms is then measured based on IC, usually at the most informative common ancestor (MICA). Finally, the similarity between two entities (e.g., genes) was derived from pairwise term similarity using best-matching based methods: average, maximum, and complete. To identify phenotypically important SNPs, we used SNPnexus (http://www.snp-nexus.org) to assess the potential significance of identified SNPs on the major transcriptome, proteome, regulatory, and structural variation models [26].

### 2.4. Targeted Next-Generation Sequencing

As proof of concept to validate the identified SNP-clusters and genes related to diabetes in our cohort, two Emirati diabetic patients (1 male and 1 female; age 60 ± 1) were selected as they have an exceptionally high level of glycated hemoglobin (HbA1c > 10.5%), fasting blood glucose values > 13 mmol/L, BMI > 40, weight 120 ± 3 kg, with dyslipidemia profile, hypertension, and obstructive sleep apnea/snoring. Extracted blood DNA was subjected to targeted DNA next-generation sequencing using the S5 semi-conductor based DNA sequencer as described previously [26]. Briefly, we used the targeted coding-exome sequencing approach by designing multiplex primers that map across key genes along the coding region of the genome. We multiplexed the two patients on one Ion Chip. A pooled barcoded amplicon-tagged library generated using Fluidigm Access Array (Fluidigm Europe B.V, Amsterdam, The Netherlands) was diluted and subjected to emulsion polymerase chain reaction (PCR) with Ion SphereTM particles with Ion Template OT2 200 Kit (Ion OneTouchTM system) following the manufacturer’s instructions (Thermo Fisher, Waltham, MA, USA). The pooled samples were sequenced using 540 Ion Chip with Ion 200 Sequencing kit on the S5 sequencer following manufacturer instructions (Thermo Fisher). The total mapped reads obtained were around 40 million per patient, with 109 mean depth and 94% uniformity coverage. 92.4% of the amplicons were aligned to the reference genome (HG19 build). The AQ20 quality mean length was 138 bp and the mean raw accuracy was 98.6%. The mean coverage across the amplicon was around ×130. The bioinformatics analysis was carried out by first aligning the data using the BWA alignment algorithm, followed by sequence filtering using SAMtools. Mutations file (VCF) was generated using vcftools. The mutations were visualized using Integrated Genome Viewer.

### 2.5. RNA-Seq from Human Islets

RNA-seq data were extracted from human pancreatic islets obtained from 75 cadaver donors (nondiabetic HbA1c < 6%, *n* = 63 and T2D/hyperglycemic HbA1c ≥ 6.3%, *n* = 12) as previously described [27,28]. RNA-seq was performed using Illumina’s TruSeq RNA Sample Preparation Kit as described previously [27,28]. Data normalization was processed using a trimmed mean of M-values and presented as Fragments/Kilobase of Exon Per Million Fragments Mapped (FPKM) or transformed into log2 counts per million using the voom-function (edgeR/limma R-packages). RNA-seq are deposited in a MIAME database (GSE50398).

### 2.6. In Silico Validation

To link the genomic SNPs to the functional and dynamic mRNA transcriptomics of the identified genes, we searched publicly available transcriptomics database “GEO Omnibus” of pancreatic islets isolated from nondiabetic and compared to diabetic samples. Then, we extracted blood transcriptomic datasets from T2D, pre-diabetic, and healthy controls. Details of the used datasets are listed in Supplementary Table S2.

### 2.7. Statistical Methods

GraphPad Prism version 7.00 for Windows (GraphPad Software, La Jolla, CA, USA) was used for statistical analysis. First, the D′Agostino–Pearson normality test was used to determine whether to perform parametric or nonparametric tests. One-way ANOVA test was performed to determine whether there are any statistically significant differences between the mean values of the controls and different groups for the gene expression and protein levels. For nonparametric tests, the Kruskal–Wallis test and Dunn’s correction for multiple testing were used. The same software was used to examine the correlations between the different parameters using linear regression. A Student’s *t*-test was used to look for the difference between two groups under a given experiment or treatment. *p* < 0.05 was considered significant.

## 3. Results

### 3.1. NHGRI-EBI GWAS Catalog Analysis Identifies Eight SNP-Clusters in Chromosome Regions with Frequent SNPs Associated with Most of the UAEDIAB-Phenotypes

**Figure 1:**
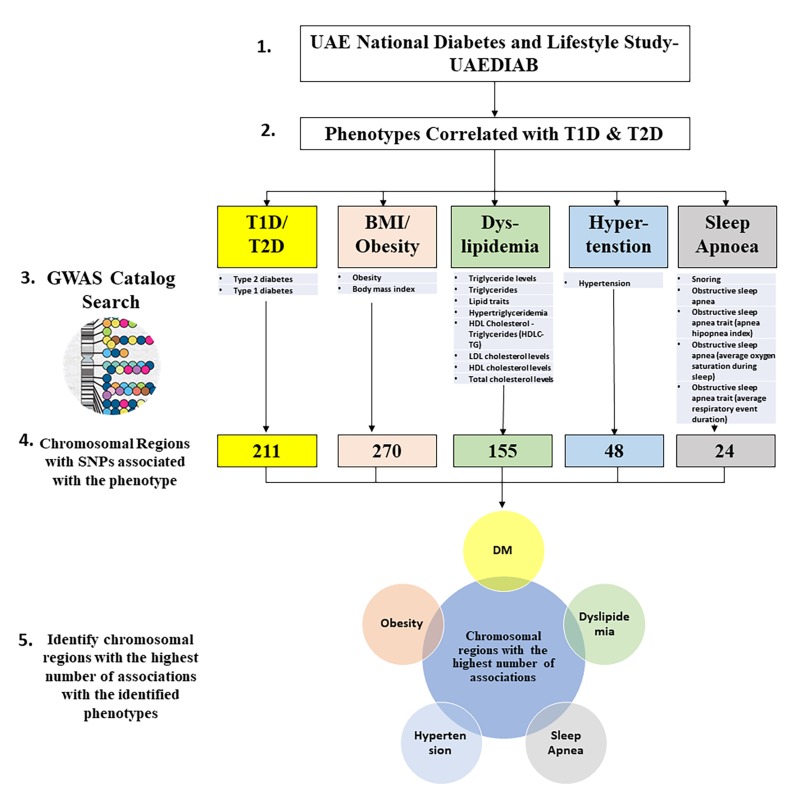
Flowchart to identify hot spots of chromosomal regions associated with single nucleotide polymorphisms (SNPs) related to the phenotypes identified using the National Human Genome Research Institute-European Bioinformatics Institute Catalog of Published Genome-Wide Association Studies (NHGRI-EBI GWAS) Catalog. T1D: type 1 diabetes; T2D: type 2 diabetes; BMI: body mass index; GWAS: Genome-Wide Association Studies; HDL: high-density lipoprotein; HDLC-TG: cholesterol-triglycerides; LDL: low-density lipoprotein; DM: diabetes mellitus.

SNPs related to the different UAEDIAB-phenotypes and diabetes were explored using the NHGRI-EBI GWAS Catalog. Our analysis showed a total of 708 chromosomal regions associated with most SNPs that have been associated with UAEDIAB-phenotypes. Of them, 211 chromosomal regions were associated with diabetes, 270 SNPs with BMI and obesity, 155 regions with lipid profile, and 48 regions with hypertension, while 24 regions were associated with obstructive sleep apnea (Figure 1).

**Figure 2:**
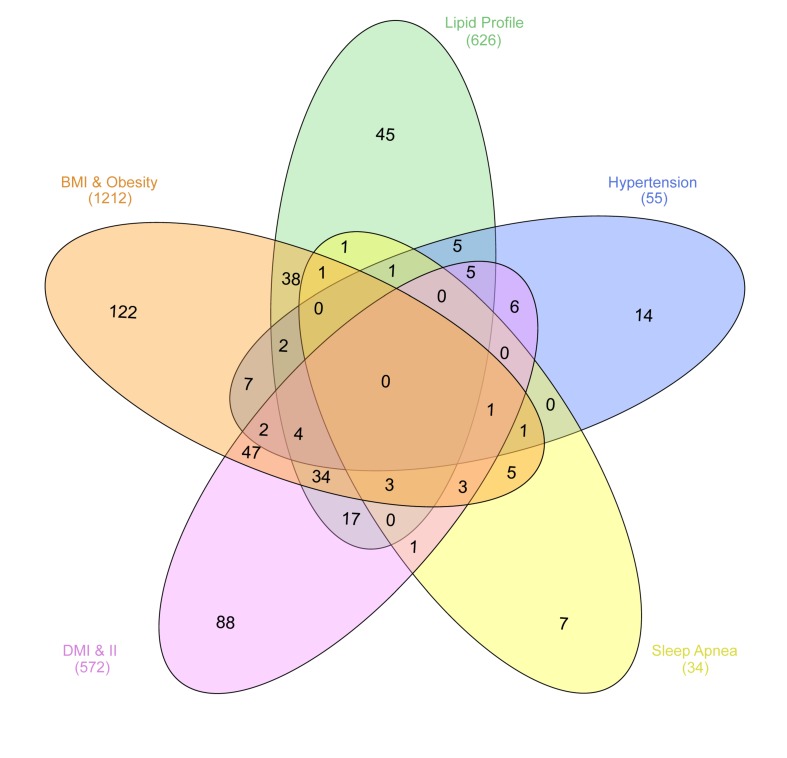
Numbers of chromosomal regions sharing SNPs correlated with the five UAEDIAB-phenotypes (BMI/obesity, dyslipidemia, hypertension, sleep apnea, and T1D/T2D). The regions were extracted from the NHGRI-EBI GWAS Catalog. The figure was generated using the InteractiVenn: a web-based tool for the analysis of sets through Venn diagrams.

Interestingly, the intersection of chromosomal regions associated with UAEDIAB-phenotypes with diabetes identified eight common chromosomal regions between the phenotypes. Of them, four chromosomal regions (8p22, 1q32.3, 12q24.13, and 7p15.2) were overlapped between BMI/obesity, dyslipidemia, hypertension, and T1D/T2D, three regions (11p11.2, 6q21, and 17q12) between BMI/obesity, dyslipidemia, sleep apnea, and T1D/T2D and only, one common region (15q26.1) was found between BMI/obesity, hypertension, sleep apnea, and T1D/T2D (Figure 2 and Table 1). Analysis of those eight shared chromosomal regions revealed 46 potential SNPs (Table 2). Data from the NHGRI-EBI GWAS Catalog revealed that 34 genes were reported to be affected by those SNPs (Table 2).

### 3.2. SNPs Enrichment Analysis Showed Significant Ontology Similarity and Metabolic Pathways Enrichment

XGR web tool was used to explore the correlation of the 46 identified SNPs as a group to each other or to UAEDIAB-phenotypes. This analysis further confirms the association between the identified 46 SNPs and the given phenotypes (FDR < 0.05). Additionally, our analysis showed a significant association between the 46 identified SNPs and other phenotypes (Table 3). Of them, 15 SNPs were associated with metabolic disease, 12 SNPs with diabetes mellitus, 13 SNPs with body mass index, and 10 SNPs with T2D.

**Figure 3:**
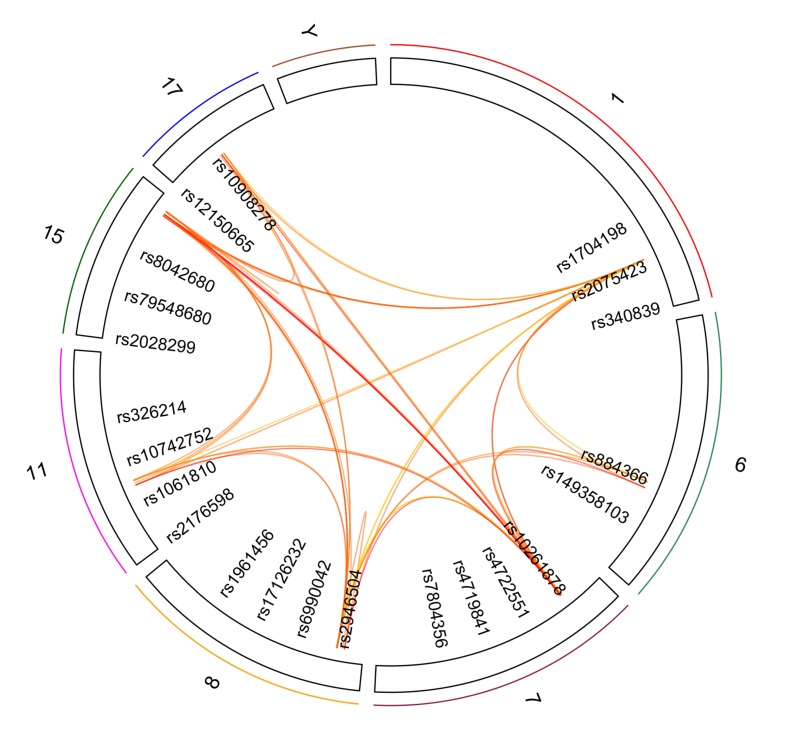
SNP-based Similarity Analysis of the identified SNPs using the “Socialiser for SNPs” option in the eXploring Genomic Relations (XGR) web tool. Seven SNPs showed similarity to at least 10 other SNPs with a similarity score of more than 0.5 in scale range from 0 to 1.

Next, we harnessed the Socialiser tool to assess the degree of relatedness or network between any two SNPs in the meaning of annotation profiles. Our results showed that 25 out of the 46 SNPs had a similarity score of more than 0.5 in scale range from 0 to 1 with at least five other SNPs (Figure 3 and Table 4). These results indicate a possible co-occurrence or functional relationship of related SNPs. Moreover, clustering of the reported 34 genes in the shared chromosomal regions according to pathway enrichment revealed a significant enrichment of four major pathways, namely developmental biology, signal transduction, and metabolism of lipids and steroids (Supplementary Table S1).

### 3.3. Targeted Next-Generation Sequencing (NGS) Confirms the Presence of Similar SNP-Clusters in Emirati Diabetic Patients

To confirm that our pipeline was able to identify similar SNP-clusters in the Emirati population, we performed NGS on two diabetic patients (as described in the methods section). As shown in Table 5 and Supplementary Figure S1, NGS analysis confirmed the presence of a high rate of SNPs (defined as more than one SNP) in the same genes identified earlier.

Out of the 34 identified genes by our pipeline, four genes (*ASAH1*, *LRP4*, *FES*, and *HSD17B12*) showed to have at least four SNPs from each patient (four was the median of the SNPs number per patients after excluding those who have one SNP only).

### 3.4. RNA-Seq Analysis in Human Pancreatic Islets Showed that HSD17B12 is Novel Candidate Gene for Pancreatic β Cell Function

**Figure 4:**
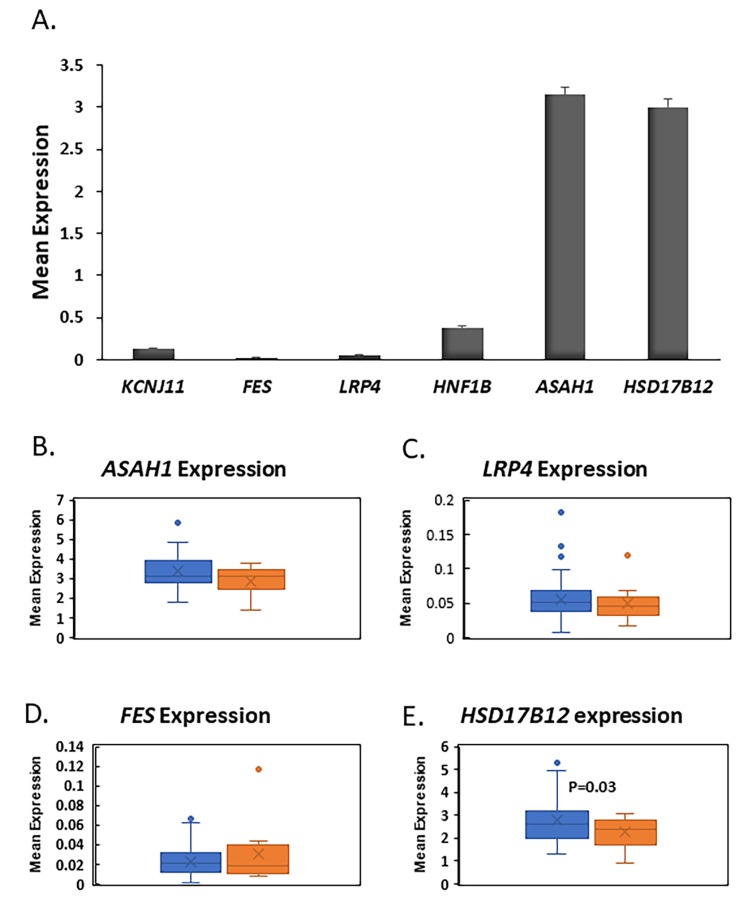
Gene expression in human pancreatic islets. (**A**) RNA-seq mean expression of *FES*, *LRP4*, ASAH1, and *HSD17B12* in non-diabetic human pancreatic islets (*n* = 63). *KCNJ11* gene was used as functional marker for pancreatic islets; (**B**–**E**) Whisker boxes of differential expression analysis of *ASAH1,* (**B**) LRP4, (**C**) *FEX*, and (**D**) *HSD17B12*; (**E**) in pancreatic islets of diabetics (*n* = 12) and non-diabetics (*n* = 63). *HSD17B12* was significantly downregulated in T2D islets (*p* = 0.03).

To gain more insights about the role of the four genes (*ASAH1*, *LRP4*, *FES*, and *HSD17B12*) in pancreatic islets, we used our published RNA-seq data to investigate their expression in human islets [27,28]. RNA-seq mean expression analysis from nondiabetic islets showed that *ASAH1* and HSD17B12 are highly expressed at a high level in human islets as compared *FES*, *LRP4*, or to the ion channel gene *KCNJ11*, a functional marker for pancreatic β-cell function. (Figure 4A). Differential expression analysis exhibited that expression of HSD17B12 is significantly reduced (*p* = 0.03) in diabetic islets (*n* = 12) when compared with nondiabetic islets (*n* = 63, Figure 4E). Expression of *ASAH1*, *FES*, and *LRP4* was not affected by diabetes status (Figure 4B–D). Collectively, based on the expression level and differential expression in human islets, our results suggest that *HSD17B12* is a candidate gene for pancreatic β-cell function.

### 3.5. Annotations of SNPs in HSD17B12

Targeted NGS performed in two local Emirati diabetic patients identified a total of four intronic SNPs in the *HSD17B12* gene (Supplementary Table S2). Interestingly, the two patients showed one common genotype of rs4573668 (homozygote C/C instead of the reference G). To identify if this SNP is phenotypically important, we used the SNPnexus tool to explore the location and functional consequences of rs4573668. Interestingly, we found that rs4573668 to be located in the 5’ untranslated region of the HSD17B12 transcript which could affect its expression, while it does not alter the amino acid sequence of the mature protein (Supplementary Figure S2). This might indicate that rs4573668 plays a role in controlling the activity and function of the *HSD17B12* in terms of transcription or epigenetic modifications.

## 4. Discussion

In this study, we employed an integrative phenotype-genotype systems approach to investigate whether UAEDIAB-phenotypes shared chromosomal regions containing SNP-clusters, which can be exploited as candidate biomarkers for pancreatic β-cell function and risk of diabetes. Our analysis identified eight shared chromosomal regions in four UAE-DIAB-phenotypes (Figure 2 and Table 1). The identified regions contain 46 SNPs with reported effect on 34 genes (Table 2), hence, putatively involved in the pathogenesis processes leading to the disease. In vivo validation on Emirati diabetic patients, showed that *ASAH1*, *LRP4*, *FES*, and *HSD17B12* have the highest number of SNPs among the 34 genes.

An important part of our study is providing more knowledge for the association of the five identified candidate genes with diabetes in human pancreatic islets from donors with and without diabetes (Figure 4). *ASAH1* gene is a member of the ceramidases family which degrade ceramide to free fatty acids and sphingosine (SPH) [29]. It has been shown that the inhibitory effects of accumulated saturated fatty acids on insulin signaling were prevented by *ASAH1* overexpression [30]. Other studies have revealed that endurance exercise was associated with a reduction in ceramide levels in the skeletal muscles of obese individuals, hence leading to the improvement of insulin sensitivity [31]. Although the expression of *ASAH1* in human islets was high, we were not able to observe any differential expression of the gene in diabetic islets (Figure 4). This suggests that ASAH1 is probably not involved in β-cell function but rather insulin signaling as evident by other reports [30,31].

*LRP4* is a single-pass transmembrane protein belonging to the LDL receptor-related protein (LRP) family. Members of this family were found to be involved in various processes, including development and physiology [32]. *LRP4* loss of function has been associated with developmental anomalies associated with Cenani–Lenz syndrome (CLS) disease, which includes limb malformation and renal agenesis [33]. Furthermore, adipocytes in LRP4 deficient mice were characterized by an improvement of glucose and insulin tolerance, lipid homeostasis, less adipocyte hypertrophy as well as reduction of serum fatty acids, thus confirming the role of *LRP4* in regulating glucose metabolism [34]. *FES* gene encodes for a non-receptor protein with tyrosine-specific activity. A recent investigation of the genetic determinants of cardiovascular diseases has found *FES* to be associated with hypertension and BMI [35]. The findings that expression of *LPR4* and *FES* is not affected by diabetes status and low expressed in human islets indicate that those genes might not be potential important players in β-cell function (Figure 4).

*HSD17B12* (Hydroxysteroid 17-β dehydrogenase) is an essential molecule in the elongation of very-long-chain fatty acids (VLCFAs) and the production of arachidonic acid through the conversion of 17-keto and 17-hydroxysteroids [36]. The gene has been recently linked to BMI and obesity susceptibility [37]. We showed that *HSD17B12* expression was very high in human islets and significantly decreased in diabetic islets compared to nondiabetic (Figure 4). The findings may suggest a role of *HSD17B12* gene in pancreatic β-cell function. In support of our RNA-seq data, it was reported that tissues involved in lipid metabolisms such as liver, kidney, muscle, and adipose tissues have a high expression level of *HSD17B12* in humans and mice [36]. Interestingly, pathway enrichment analyses of the 34 genes pointed out that *HSD17B12* is involved in signal transduction and metabolism of lipids and steroids, which fits well with studies of phenotypes.

A previous study showed that individuals carrying the T2D risk allele T for the intronic SNP rs11037579 had lower expression of *HSD17B12* in adipose tissue of insulin-resistant subjects [38]. Additionally, rs1061810 was documented to be associated with T2D indicating a role for *HSD17B12* in diabetes [39]. Our results indicated that genotyping of rs4573668 (C/C) is common in the local Emirati patients. Since the rs4573668 variant might lead to a change in the protein amino acid sequence, therefore, it could play a significant role in controlling the activity and function of the *HSD17B12* in terms of transcription, translation, and epigenetic modifications. Moreover, the findings that *HSD17B12* expression is reduced in diabetic islets, highlights the potential of utilization of this gene as an early biomarker in blood samples for pre-diabetic patients.

In summary, our study proposes HSD17B12 as a causal gene for T2D and pancreatic β-cell function. More future functional studies still needed to validate the finding.

## 5. Conclusions

In conclusion, our combined approach supports the existence of common chromosomal regions and SNP-clusters among obesity/BMI, hypertension, obstructive sleep apnea, dyslipidemia, and diabetes which might be involved in the pathogenesis of these clinically related phenotypes.

We were able to replicate these SNP-clusters on the local Emirati population different from the ethnic groups available in GWAS. *HSD17B12* was identified as a candidate gene for β-cell function. Our data are the implementation of genomics-based approaches to chronic disease detection using bioinformatics in silico approach, and, therefore, further functional validation is still needed to elucidate the role of *HSD17B12* and rs4573668 in the pathogenesis of T2D. In the end, we believe that such knowledge could increase our understanding of diabetes and facilitate the development of drugs.

## Figures and Tables

**Table 1:** List of overlapped chromosomal regions that correlated with UAEDIAB-phenotypes and diabetes.

Number of Shared Phenotypes	Shared Phenotypes	Number of Shared Regions	Shared Regions/Bands
4 Phenotypes	(BMI/obesity) and (dyslipidemia) and (hypertension) and (T1D/T2D)	4	8p22,1q32.3,12q24.13,7p15.2
	(BMI/obesity) and (dyslipidemia) and [sleep apnea] and (T1D/T2D)	3	11p11.2,6q21,17q12
	(BMI/obesity) and (hypertension) and (sleep apnea) and (T1D/T2D)	1	15q26.1
3 Phenotypes	(BMI/obesity) and (dyslipidemia) and (T1D/T2D)	34	16q12.2,19q13.32,16p11.2,5q13.3,11p15.4, 10p13,16q23.2,18q21.32,18q11.2,6p22.3, 2p23.3,19q13.11,10p15.1,22q12.3,15q15.1, 1p31.3,19p13.2,2q36.3,11p15.1,12q24.12, 12q24.11,11q13.1,2q24.3,1p32.3,1q21.3, 10q25.2,6p21.32,6p21.1,6q23.3,17p13.2, 10q21.3,12q24.31,3p25.2,3q21.1
	(dyslipidemia) and (hypertension) and (T1D and T2D)	5	10q23.33,1q41,1q43,6p21.33,8q24.12
	(BMI/obesity) and (hypertension) and (T1D/T2D)	2	16p12.3,1p13.2
	(BMI/obesity) and (sleep apnea) and (T1D/T2D)	3	1q32.1,1q42.2,11q13.4
2 Phenotypes	(dyslipidemia) and (T1D/T2D)	17	6q13,6q27,20q13.12,1q42.13,5q11.2,4p16.3, 6q24.1,12p13.31,9q34.2,8q24.3,17p13.1, 9p24.2,22q12.2,1p34.3,7q32.2,1p22.1,2q33.2
	(BMI/obesity) and (T1D/T2D)	47	6q23.1,10q22.3,8q21.13,2p25.3,4p12, 12q13.12,14q31.1,3q27.2,2p16.1,9p21.1,4q28.2, 17q21.32,18q12.3,1p21.3,9q22.31,15q14, 9q22.2,5q33.2,14q11.2,3p14.1,12q12,8q22.3, 21q22.3,2p23.2,17p11.2,2q21.3,9q31.3, 10q26.13,17p13.3,11p15.5,11q13.3,8q24.21, 7q36.3,1p12,3q23,6p21.2,7p12.1,9p24.1, 14q24.1,18p11.21,13q31.1,5q21.1,2p21,12p12.1, 7p14.3,8q22.1,9p21.3
	(hypertension) and (T1D/T2D)	6	5q31.1,18p11.31,7q22.1,21q22.11,8p11.21, 9q21.32
	(sleep apnea) and (T1D/T2D)	1	2p24.3
1 Phenotype	(T1D/T2D)	89	8q24.11,11q14.3,4q35.1,6q12,17q21.33, 3q26.2,17q11.2,4q22.2,22q13.33,7p21.2,5q22.2, 3q13.31,11p12,15q22.2,2q23.3,11q24.3, 3q26.33,1q32.2,10q26.3,19q13.2,4q32.3, 14q32.2,2q24.2,7q32.1,9q34.3,3p24.3,Xq28, 2q33.1,3q27.3,13q21.31,6q25.1,13q14.13, 13q21.33,13q22.1,16p13.12,20q11.21,1q21.2, 5q14.2,20p12.2,2q14.3,14q23.1,15q24.3, 12q13.2,1p22.3,6q15,16p13.13,18q22.2,2q11.2, 5p13.2,15q25.1,4q27,4p15.2,6q22.32,10q23.31, 17q21.1,17q21.2,20p13,13q22.2,7p15.1, 10q24.2,10q26.11,13q12.12,8q24.22,11p15.4, 11p15.5,3p23,12q21.2,1p22.2,12q21.1,2q12.1,10q22.1, 3q12.3,9p23,9q21.31,12q14.3, 4p16.1,4q31.3,6p24.3,13q12.13,12p11.22, 2p23.1,7p14.1,8q13.2,12p11.21,2p16.2, 10q26.12,16q24.1,3p14.3,20q13.31

**Table 2:** List of the 46 SNPs located in the identified eight shared chromosomal regions and their reported affected genes. Data extracted from the NHGRI-EBI GWAS Catalog.

SNP ID	Mapped Gene	Chro. Region	Location	Reported Gene
rs3817334	*MTCH2*	11p11.2	11:47629441	*MTCH2*
rs7124681	*CELF1*	11p11.2	11:47508395	*CUGBP1*
rs11066280	*HECTD4*	12q24.13	12:112379979	*HECTD4*
rs4430796	*HNF1B*	17q12	17:37738049	*HNF1B*
rs2176598	*HSD17B12*	11p11.2	11:43842728	*HSD17B12*
rs17696736	*NAA25*	12q24.13	12:112049014	Not Reported
rs2028299	*AP3S2*	15q26.1	15:89831025	*AP3S2*
rs9400239	*FOXO3*	6q21	6:108656460	*FOXO3*
rs17126232	*AC124242.3*	8p22	8:18120141	*ASAH1*
rs6990042	*SGCZ*	8p22	8:14316465	*SGCZ*
rs10742752	*AC103855.3*	11p11.2	11:45416824	*SYT13*
rs17630235	*TRAFD1*, *HECTD4*	12q24.13	12:112153882	Not reported
rs1439620	*AC013394.1*, *LINC01578*	15q26.1	15:92886416	*LOC100507217*
rs12150665	*GGNBP2*	17q12	17:36558947	*GGNBP2*
rs3800229	*FOXO3*	6q21	6:108675760	*FOXO3*
rs35424364	*CCDC162P*	6q21	6:109322403	*C6ORF183*, *CCDC162P*
rs1495741	*PSD3*, *NAT2*	8p22	8:18415371	*NAT2*
rs10838738	*MTCH2*	11p11.2	11:47641497	*MTCH2*
rs326214	*MADD*	11p11.2	11:47276809	*LRP4*
rs74472562	*TSPAN18*	11p11.2	11:44741205	*RP11-45A12.2*, *TSPAN18*
rs1061810	*HSD17B12*, *AC087521.2*, *AC087521.4*	11p11.2	11:43856384	*HSD17B12*
rs936674	*AC091078.1*	15q26.1	15:93360368	*RP11-266O8.1*
rs148024591	*AC091078.1*	15q26.1	15:93371222	*RP11-266O8.1*
rs2521501	*FES*	15q26.1	15:90894158	*FURIN, FES*
rs8042680	*PRC1*, *PRC1-AS1*	15q26.1	15:90978107	*PRC1*
rs12899811	*VPS33B*	15q26.1	15:91000846	*PRC1*
rs79548680	*RCCD1*	15q26.1	15:90962549	*PRC1*
rs1877031	*STARD3*	17q12	17:39657827	*STARD3*
rs4796285	*AC243830.1*, *LHX1-DT*	17q12	17:36824731	Not reported
rs10908278	*HNF1B*	17q12	17:37739961	*HNF1B*, *TCF2*
rs1704198	*AC096639.1*	1q32.3	1:213737151	*PROX1*
rs340839	*PROX1*	1q32.3	1:213988477	*PROX1*
rs7526425	*AC105275.1*, *SLC30A1*, *RD3*	1q32.3	1:211527316	*SLC30A1*
rs2075423	*PROX1-AS1*	1q32.3	1:213981376	*PROX1*
rs884366	*CCDC162P*	6q21	6:109252892	*LOC100996634*
rs149358103	*RPS27AP11*, *LINC02541*	6q21	6:11358684	*SOCS5P5*, *MARCKS*
rs10261878	*AC010719.1*, *AC018706.1*	7p15.2	7:25910925	*NFE2L3*, *MIR148A*
rs4719841	*NFE2L3*, *MIR148A*	7p15.2	7:25957916	*MIR148A*
rs4722551	*NFE2L3*, *MIR148A*	7p15.2	7:25952206	*MIR148A*
rs6969780	*HOXA3*, *HOXA-AS2*	7p15.2	7:27119517	*HOXA3*
rs10279895	*HNRNPA1P73*, *RPL35P4*	7p15.2	7:27288591	*EVX1*, *HOXA*
rs7804356	*SKAP2*	7p15.2	7:26852046	Not reported
rs4921914	*PSD3*, *NAT2*	8p22	8:18414928	*NAT2*
rs1961456	*NAT2*	8p22	8:18398199	*NAT2*
rs115706913	*SGCZ*	8p22	8:14224308	Not Reported
rs2946504	*TRMT9B*	8p22	8:12954071	*KIAA1456*

**Table 3:** Enrichment analysis of the 46 SNPs found in eight shared chromosomal regions in at least four of the UAEDIAB-phenotypes.

	Term Name	Number of SNPs Overlapped	SNP IDs	Z-Score	*p*-Value	FDR
**1**	Metabolic Disease	15	rs1061810, rs10908278, rs11066280, rs12899811, rs149358103, rs1704198, rs17126232, rs17696736, rs2028299, rs2075423, rs2946504, rs4430796, rs7804356, rs79548680, rs8042680	13.2	8.20 × 10^−15^	2.60 × 10^−13^
**2**	Diabetes Mellitus	12	rs1061810, rs10908278, rs12899811,rs149358103, rs17696736, rs2028299, rs2075423, rs2946504, rs4430796, rs7804356, rs79548680, rs8042680	12.2	1.50 × 10^−12^	2.30 × 10^−11^
**3**	Body Mass Index	13	rs10261878, rs10742752, rs10838738, rs12150665, rs1439620, rs17630235, rs2176598, rs3800229, rs3817334, rs6990042, rs7124681, rs936674, rs9400239	10.9	6.40 × 10^−12^	4.80 × 10^−11^
**4**	Sleep Apnea	4	rs148024591, rs35424364, rs4796285, rs74472562	29.7	7.80 × 10^−12^	4.80 × 10^−11^
**5**	Sleep Apnea	4	rs148024591, rs35424364, rs4796285, rs74472562	29.7	7.80 × 10^−12^	4.80 × 10^−11^
**6**	Type II Diabetes Mellitus	10	rs1061810, rs10908278, rs12899811, rs2946504, rs149358103, rs8042680, rs2028299, rs2075423, rs4430796, rs79548680	11.9	2.30 × 10^−11^	1.20 × 10^−10^
**7**	Sleep Apnea Measurement	4	rs148024591, rs35424364, rs4796285, rs74472562	20.5	3.90 × 10^−10^	1.70 × 10^−9^
**8**	Sleep Disorder	4	rs148024591, rs35424364, rs4796285, rs74472562	15.5	6.50 × 10^−9^	2.50 × 10^−8^
**9**	Hypertension	5	rs10279895, rs11066280, rs115706913, rs2521501, rs6969780	12.1	1.40 × 10^−8^	4.80 × 10^−8^
**10**	Triglyceride Measurement	6	rs11066280, rs1495741, rs340839, rs4719841, rs4722551, rs4921914	7.9	5.70 × 10^−7^	1.8 × 10^−6^
**11**	Lipid Measurement	9	rs11066280, rs1495741, rs1877031, rs326214, rs340839, rs4719841, rs4722551, rs4921914, rs884366	6.21	1.9 × 10^−6^	5.4 × 10^−6^
**12**	Lipoprotein Measurement	7	rs11066280, rs1495741, rs1877031, rs1961456, rs326214, rs4722551, rs884366	5.45	1.9 × 10^−5^	0.00005
**13**	Diastolic Blood Pressure	5	rs10279895,rs11066280, rs17696736, rs2521501, rs6969780	5.5	4.1 × 10^−5^	9.7 × 10^−5^
**14**	Mean Arterial Pressure	3	rs17696736, rs2521501, rs6969780	6.2	5.3 × 10^−5^	0.00012
**15**	Physical Activity Measurement	3	rs3800229, rs3817334, rs7124681	5.31	0.00015	0.00028
**16**	High-Density Lipoprotein Cholesterol Measurement	4	rs11066280, rs1877031, rs326214, rs884366	4.92	0.00015	0.00028
**17**	Drinking Behavior	3	rs11066280, rs17696736, rs2521501	5.27	0.00016	0.00028
**18**	Obesity	2	rs1704198, rs17126232	5.4	0.00025	0.00043
**19**	Systolic Blood Pressure	4	rs11066280, rs17696736, rs2521501, rs6969780	4.21	0.00046	0.00074
**20**	Parental Longevity	2	rs17630235, rs17696736	4.37	0.00073	0.0011
**21**	Total Cholesterol Measurement	3	rs1495741, rs1961456, rs4722551	4.01	0.00081	0.0012
**22**	Type I Diabetes Mellitus	2	rs17696736, rs7804356	4.26	0.00083	0.0012
**23**	Blood Pressure	5	rs10279895, rs11066280, rs17696736, rs2521501, rs6969780	3.68	0.00089	0.0012
**24**	Behavior	5	rs11066280, rs17696736, rs2521501, rs3817334, rs9400239	3.61	0.001	0.0013
**25**	Alcohol Drinking	2	rs17696736, rs2521501	3.54	0.0019	0.0023
**26**	Longevity	2	rs17630235, rs17696736	3.53	0.002	0.0023
**27**	Blood Metabolite Measurement	2	rs1495741, rs4921914	3.53	0.002	0.0023
**28**	Vital Signs	5	rs10279895, rs11066280, rs17696736, rs2521501, rs6969780	3.1	0.0026	0.0029
**29**	Smoking Behavior	2	rs3817334, rs9400239	1.71	0.026	0.028

**Table 4:** List of identified SNPs with related genes that showed similarity to each other using the Socialiser tool.

SNP	Gene Related to the SNP	Number of SNPs with Similarity Score > 0.5 in a Scale of 0–1
rs11066280	*ALDH2*	23
rs17696736	*C12orf30*	15
rs17126232	*ASAH1*	13
rs1704198	*PROX1*	13
rs9400239	*FOXO3*	10
rs3800229	*FOXO3*	9
rs1961456	*NAT2*	9
rs1495741	*NAT2*	9
rs4722551	*MIR148A*	8
rs884366	*LOC100996634*	7
rs326214	*LRP4*	7
rs1877031	*STARD3*	7
rs4719841	*MIR148A*	6
rs340839	*PROX1*	6
rs2028299	*AP3S*	5
rs10908278	*HNF1B*, *TCF2*	5
rs1061810	*HSD17B12*	5
rs2946504	*KIAA1456*	5
rs4921914	*NAT2*	5
rs12899811	*PRC1*	5
rs79548680	*PRC1*	5
rs8042680	*PRC1*	5
rs2075423	*PROX1*	5
rs2075423	*PROX1*	5
rs149358103	*SOCS5P5*, *MARCKS*	5

**Table 5:** List of SNPs identified by next-generation sequencing (NGS) from two diabetic patients in genes located in the shared chromosomal region.

Identified Genes	Number of SNPs per Sample	
	Patient 1	Patient 2
*ASAH1*	20	16
*LRP4*	8	7
*FES*	9	6
*HSD17B12*	7	4
*HNF1B*	4	4
*HECTD4*	2	6
*SH2B3*	4	4
*NAT2*	4	2
*SGCZ*	3	1
*FURIN*	3	1
*GGNBP2*	5	1
*TSPAN18*	2	2
*ALDH2*	1	1
*STARD3*	1	1
*MTCH2*	1	2
*NDUFS3*	1	1
*PRC1*	0	0
*PTPN11*	1	0
*CELF1*	3	0
*NAA25*	2	0

## Supplementary Materials

The following are available online at https://www.mdpi.com/2073-4425/11/4/461/s1, Figure S1: Screenshots of examples SNPs in the genes identified generated by targeted DNA next-generation sequencing in Emirati diabetic patients, Figure S2: List of the location and functional consequences of rs4573668 as predicted by SNPnexus tool, Table S1: Enriched Ontology Clusters of the identified 34 genes associated with the UAEDIA-phenotypes, Table S2: List of the 4 identified SNPs in *HSD17B12* gene in two Emirati diabetic patients, with their location and significance in gene expression.
