# Supplementary material for: An Integrative Phenotype–Genotype Approach Using Phenotypic Characteristics from the UAE National Diabetes Study Identifies HSD17B12 as a Candidate Gene for Obesity and Type 2 Diabetes
Source: Genes (Basel). 2020 Apr 23;11(4):461. doi: 10.3390/genes11040461 (PMC7230604; doi:10.3390/genes11040461)
Supplement: Supplementary file 1 [file genes-11-00461-s001.pdf]

Supplementary information:

Supplementary Figure 1. Screenshots of examples SNPs in the genes identified generated by targeted DNA next-generation sequencing in Emirati diabetic patients. Mutations generated using vcftools and visualized using IGV.

A- ASAH1

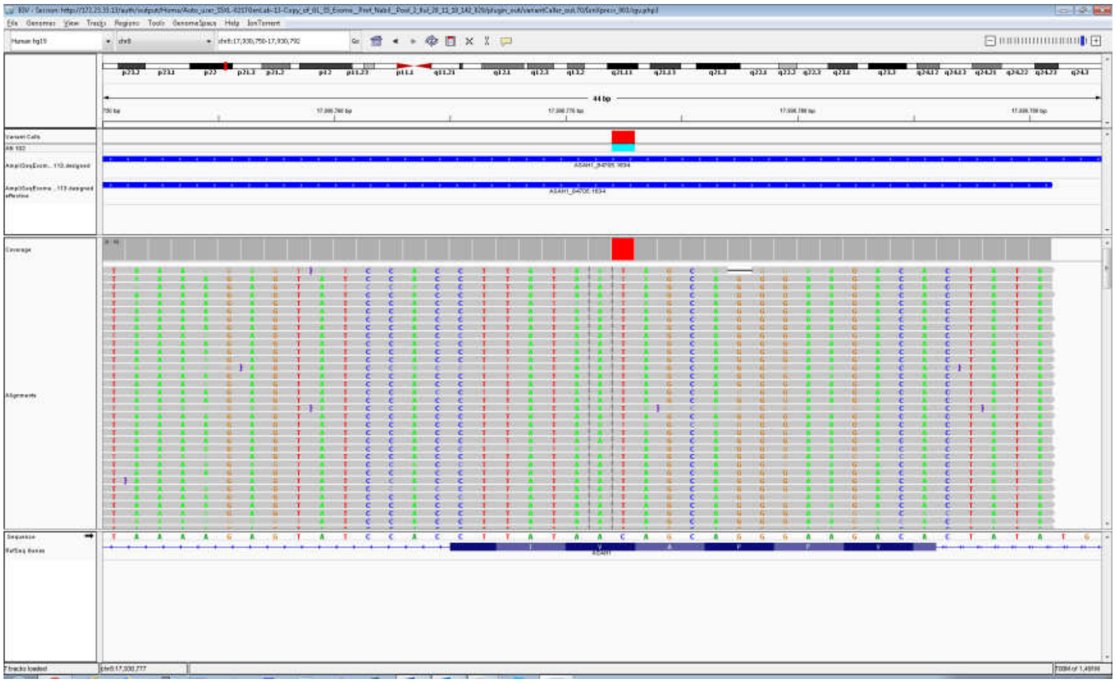

B- FES

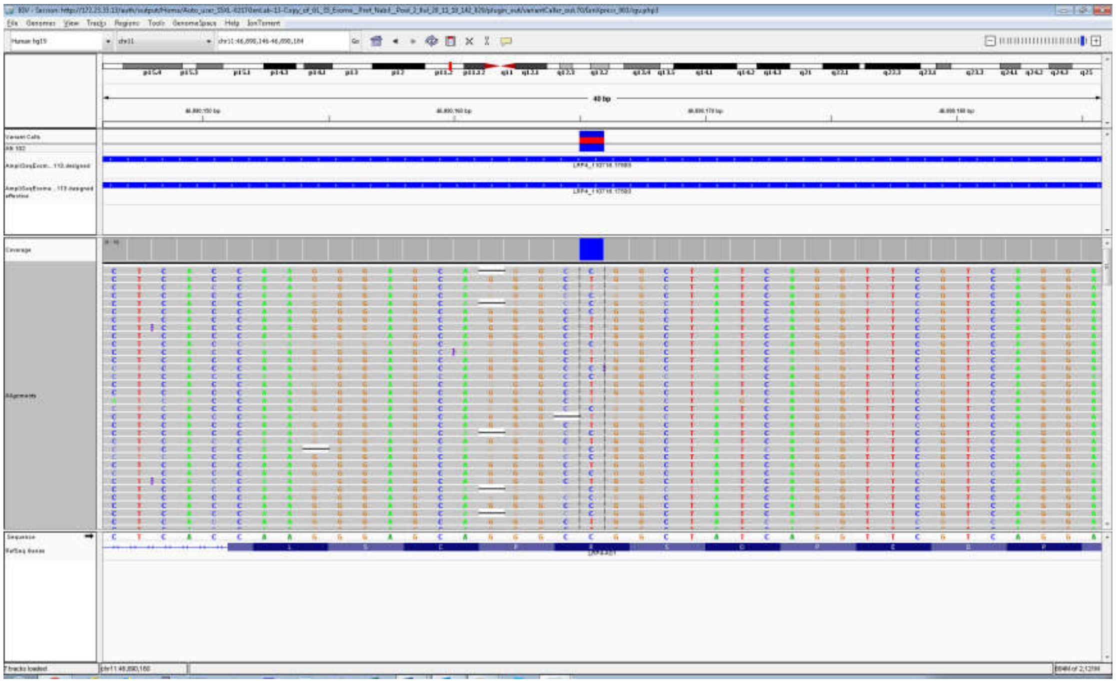

C- LPR4

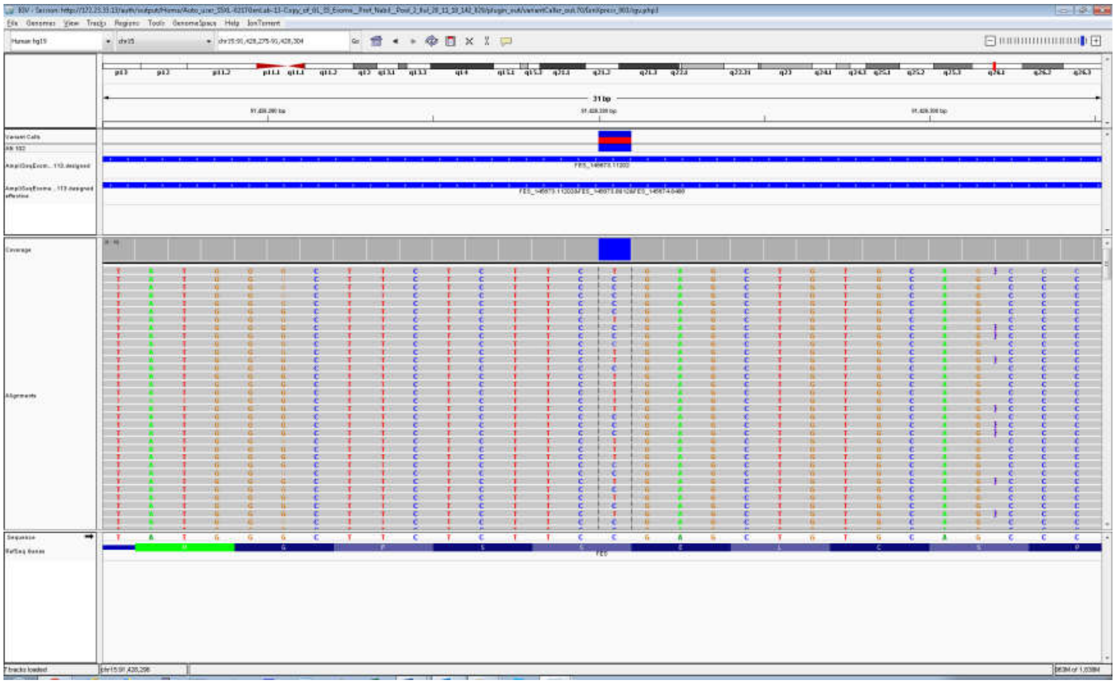

D- HSD17B12

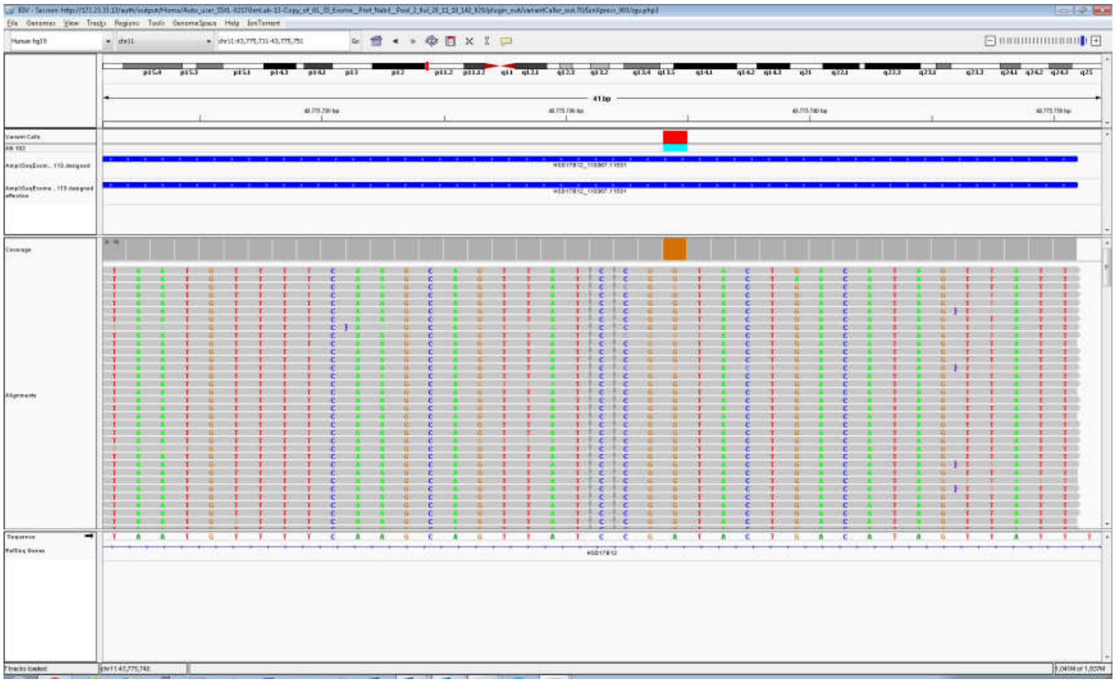

Supplementary Figure 2; List of the location and functional consequences of rs4573668 as predicted by SNPnexus tool.

<table>
<tr><th>Variation ID</th><th>Chromosome</th><th>Position</th><th>Variant</th><th>Strand</th><th>Symbol</th><th>Gene</th><th>Transcript</th><th>Predicted Function</th><th>CDNA Position</th><th>CDS Position</th><th>AA Position</th><th>AA Change</th><th>Detail</th><th>Splice Distance</th><th>Proteins</th></tr>
<tr><td>rs4573668</td><td>chr11</td><td>43681065</td><td>G|C</td><td>1</td><td>HSD17B12</td><td>ENSG00000149084</td><td>ENST0000637401</td><td>intronic</td><td>None</td><td>None</td><td>None</td><td>None</td><td>None</td><td>78</td><td>None</td></tr>
<tr><td>rs4573668</td><td>chr11</td><td>43681065</td><td>G|C</td><td>1</td><td>HSD17B12</td><td>ENSG00000149084</td><td>ENST0000531185</td><td>intronic</td><td>None</td><td>None</td><td>None</td><td>None</td><td>None</td><td>78</td><td>None</td></tr>
<tr><td>rs4573668</td><td>chr11</td><td>43681065</td><td>G|C</td><td>1</td><td>HSD17B12</td><td>ENSG00000149084</td><td>ENST0000636007</td><td>intronic</td><td>None</td><td>None</td><td>None</td><td>None</td><td>None</td><td>78</td><td>None</td></tr>
<tr><td>rs4573668</td><td>chr11</td><td>43681065</td><td>G|C</td><td>1</td><td>AC068205.2</td><td>ENSG00000283341</td><td>ENST0000532864</td><td>non-coding intronic</td><td>None</td><td>None</td><td>None</td><td>None</td><td>None</td><td>40031</td><td>None</td></tr>
<tr><td>rs4573668</td><td>chr11</td><td>43681065</td><td>G|C</td><td>1</td><td>AC068205.2</td><td>ENSG00000283341</td><td>ENST0000529261</td><td>non-coding intronic</td><td>None</td><td>None</td><td>None</td><td>None</td><td>None</td><td>7926</td><td>None</td></tr>
<tr><td>rs4573668</td><td>chr11</td><td>43681065</td><td>G|C</td><td>1</td><td>HSD17B12</td><td>ENSG00000149084</td><td>ENST0000638034</td><td>Supstream</td><td>None</td><td>None</td><td>None</td><td>None</td><td>None</td><td>None</td><td>None</td></tr>
<tr><td>rs4573668</td><td>chr11</td><td>43681065</td><td>G|C</td><td>1</td><td>HSD17B12</td><td>ENSG00000149084</td><td>ENST0000527433</td><td>intronic</td><td>None</td><td>None</td><td>None</td><td>None</td><td>None</td><td>261</td><td>None</td></tr>
<tr><td>rs4573668</td><td>chr11</td><td>43681065</td><td>G|C</td><td>1</td><td>HSD17B12</td><td>ENSG00000149084</td><td>ENST0000636722</td><td>coding</td><td>271</td><td>238</td><td>80</td><td>V>L</td><td>nonsyn</td><td>None</td><td>MESALPAAGFLYW
VGAGTVAYLALRIS
YSLFTALRVWGVG
NEAGVGPGLGEW
AGESDAAPRPRSR
GGPDQAWAVMTS
SGVCSWPSPPQLS
SFSGSCAPRAPPGS
LGCLLLA*|MESAL
PAAGFLYWVGAGT
VAYLALRISYSLFTA
LRVWGVGNEAGV
GPGLGEWAGESDA
APRPRSRGGPDQA
WAVMTSSGLCSW
PSPPQLSSFSGSCA
PRAPPGSLGCLLLA
*</td></tr>
<tr><td>rs4573668</td><td>chr11</td><td>43681065</td><td>G|C</td><td>1</td><td>HSD17B12</td><td>ENSG00000149084</td><td>ENST00000278353</td><td>intronic</td><td>None</td><td>None</td><td>None</td><td>None</td><td>None</td><td>78</td><td>None</td></tr>
<tr><td>rs4573668</td><td>chr11</td><td>43681065</td><td>G|C</td><td>1</td><td>HSD17B12</td><td>ENSG00000149084</td><td>ENST00000395700</td><td>intronic</td><td>None</td><td>None</td><td>None</td><td>None</td><td>None</td><td>78</td><td>None</td></tr>
</table>

Supplementary Table 1: Enriched Ontology Clusters of the identified 34 genes associated with the UAEDIA-phenotypes.

Pathway Id	Pathway Description	Parent Description	Genes	Gene Count
R-HSA-1266738	Developmental Biology	Developmental Biology	HNF1B,FES,FOXO3,HOXA3	4
R-HSA-162582	Signal Transduction	Signal Transduction	FES,MADD,FOXO3,PRC1	4
R-HSA-1430728	Metabolism	Metabolism	HSD17B12,STARD3,NAT2	3
R-HSA-1433557	Signaling by SCF-KIT	Signal Transduction	FES,FOXO3	2
R-HSA-196071	Metabolism of steroid hormones	Metabolism	HSD17B12,STARD3	2
R-HSA-556833	Metabolism of lipids	Metabolism	HSD17B12,STARD3	2
R-HSA-8957322	Metabolism of steroids	Metabolism	HSD17B12,STARD3	2

Supplementary Table 2. List of the 4 identified SNPs in HSD17B12 gene in two Emirati diabetic patients, with their location and significance in gene expression.

ID	SNP	Chromosome	Position	Overlapped Gene	Annotation
chr11:43681065:G/C:1	rs4573668	chr11	43681065	HSD17B12	intronic,coding nonsyn,5upstream
chr11:43750999:A/G:1	rs12801203	chr11	43750999	HSD17B12	intronic,non-coding intronic
chr11:43754184:A/G:1	rs4643069	chr11	43754184	HSD17B12	intronic,non-coding intronic,3downstream
chr11:43830941:T/C:1	rs6485471	chr11	43830941	HSD17B12	intronic,non-coding intronic,non-coding
